# Nrf2 and Redox Status in Prediabetic and Diabetic Patients

**DOI:** 10.3390/ijms151120290

**Published:** 2014-11-06

**Authors:** Angélica S. Jiménez-Osorio, Alejandra Picazo, Susana González-Reyes, Diana Barrera-Oviedo, Martha E. Rodríguez-Arellano, José Pedraza-Chaverri

**Affiliations:** 1 Faculty of Chemistry, Department of Biology, National Autonomous University of Mexico (UNAM), University City 04510 DF, Mexico; E-Mails: joas17@hotmail.com (A.S.J.-O.); suxan05@gmail.com (S.G.-R.); 2 Faculty of Medicine, Department of Pharmacology, National Autonomous University of Mexico (UNAM), University City 04510 DF, Mexico; E-Mails: alepicazo1980@gmail.com (A.P.); dianabarrera@hotmail.com (D.B.-O.); 3 Research Department, Hospital Regional “Lic. Adolfo López Mateos”, ISSSTE, Av. Universidad 1321, Florida 01030 DF, Mexico; E-Mail: marthaeunicer@yahoo.com.mx

**Keywords:** Nrf2, prediabetes, diabetes, oxidative stress, glycated hemoglobin

## Abstract

The redox status associated with nuclear factor erythroid 2-related factor-2 (Nrf2) was evaluated in prediabetic and diabetic subjects. Total antioxidant status (TAS) in plasma and erythrocytes, glutathione (GSH) and malondialdehyde (MDA) content and activity of antioxidant enzymes were measured as redox status markers in 259 controls, 111 prediabetics and 186 diabetic type 2 subjects. Nrf2 was measured in nuclear extract fractions from peripheral blood mononuclear cells (PBMC). Nrf2 levels were lower in prediabetic and diabetic patients. TAS, GSH and activity of glutamate cysteine ligase were lower in diabetic subjects. An increase of MDA and superoxide dismutase activity was found in diabetic subjects. These results suggest that low levels of Nrf2 are involved in the development of oxidative stress and redox status disbalance in diabetic patients.

## 1. Introduction

Diabetes mellitus (DM) is an epidemic disease that is considered a health problem around the world. According to the International Diabetes Federation in 2013, 381 million people suffered from diabetes, and it is estimated that it will be almost double by 2030 [1]. Almost 6.4 million Mexican adults with diabetes were identified by the 2012 National survey, which represents about 9.2% of adults in Mexico with diabetes type 2 (DM2) [2,3]. DM2 diagnosis is based on fasting plasma glucose (FPG; >126 mg/dL or 7.0 mmol/L) and glycated hemoglobin (HbA1c; >6.5%). Furthermore the identification and diagnosis of prediabetic states (FPG: 100–125 mg/dL or 5.5–6.9 mmol/L; HbA1c: 5.7%–6.4%) [4] are crucial to understand the development of the disease.

DM is a multifactorial disease that includes genetic and environmental factors and the role of oxidative stress in this pathology was recognized two decades ago. Studies have shown that oxidative stress is associated with beta cell dysfunction [5] and the development of insulin resistance [6,7]. Diabetic patients from these studies showed a decrease in antioxidant levels, low activity of antioxidant enzymes, and an increase in both reactive oxygen species (ROS) production and oxidative stress markers. In addition, hyperglycemia can induce the overproduction of superoxide anions that affect several pathways that leads to diabetic complications [8,9]. Oxidative stress is often seen as an imbalance resulting from altered gene expression. The transcription factor called nuclear factor (erythroid-derived 2)-like 2 or Nrf2 is referred to as the “master regulator” of the antioxidant response; it modulates the expression of a considerable number of genes, including not only those that control antioxidant enzymes, but also genes that control immune and inflammatory responses [10]. Under non-stress conditions, concentrations of Nrf2 are low, and the protein is retained in the cytoplasm owing to its association with Kelch-like ECH-associated inhibitor 1 (Keap1), also called Nrf2 inhibitor. In response to oxidative stress, a complex series of events leads to the stabilization of Nrf2 and its translocation into the nucleus [11], where it upregulates the expression of several genes whose promoter region contains an antioxidant response element (ARE) [12]. The relationship between Nrf2 and diabetes has been evaluated. In animal models, it has been shown that insulin signaling is necessary for Nrf2 activation [13]. When Nrf2 is activated, it protects β-cells from damage, and prevents the onset of diabetes [14,15], increasing insulin sensitivity [16,17], which can lead to a better glucose control [13,18] and prevention of the development of diabetic micro and macro vascular complications [19,20,21,22]. However, the way that Nrf2 exerts its anti-diabetic action is not fully understood at clinical level. Besides, by ethical constraints, it is inaccessible to use many methodologies in clinical trials. Thus, Nrf2 was measured in nuclear fractions of peripheral blood mononuclear cells (PBMC) from prediabetic and diabetic patients to establish the role of Nrf2 in diabetes. Total antioxidant status (TAS), glutathione (GSH) and malondialdehyde (MDA) were measured as oxidative markers and the activity of glutathione peroxidase (GPx), glutathione reductase (GR), γ-glutamate cysteine ligase (GCL) and Cu-Zn-superoxide dismutase (SOD-1) were chosen to describe the redox status.

## 2. Results

### 2.1. Clinical and Anthropometrical Characteristics

A total of 559 subjects were involved in the study, with 260 control (CTRL), 116 prediabetic (PRE) and 183 DM2 subjects. Characteristics of clinical and anthropometrical values are shown in Table 1. There is an adverse lipid profile and presence of insulin resistance measured by homeostasis model assessment of insulin resistance (HOMA-IR) since prediabetic status. Significant differences in the DM2 group were found in age, body mass index (BMI), systolic blood pressure (SBP), diastolic blood pressure (DBP), glucose, HbA1c, triglycerides (TG) and high-density lipoprotein-cholesterol (HDL-C). Therefore, these parameters are included in the multivariated analysis as possible potential confounders in the redox response. The population sample was representative of Mexico in overweight terms. In fact, the average of BMI is in obesity ranges in the prediabetic subjects and is lower in diabetic group (31.14 *vs.* 29.14 kg·m^2^). Systolic and diastolic pressures are altered in the DM2 group, with an increment of the average between 5.08% and 3.24%. From the CTRL group, 26.6% reported with diagnosed hypertension and 15.2% and 58.2% for PRE and DM2 subjects, respectively. As expected, fasting glycemia and HbA1c are altered in prediabetic and diabetic status (average increase of glucose: 15% for PRE and 41% for DM2, and for HbA1c: 22% for PRE and 30% for DM2, each *vs.* CTRL). TG levels increase during prediabetic status and are higher in diabetes. Of these prediabetic and diabetic subjects, 26.7% and 23%, respectively, reported being on treatment. HDL-C was found lower in PRE and DM2 subjects when compared with the CTRL group (9.8% and 13.7%, respectively lower than CTRL), no changes were observed in total cholesterol (T-C) and low density lipoprotein-cholesterol (LDL-C).

**Table 1:** Clinical and anthropometrical characteristics of control (CTRL), prediabetic (PRE) and diabetes mellitus (DM2) individuals. Data are presented as mean (interquartile range). Statistical significance *p* < 0.05: ^a^ *vs.* CTRL and ^b^ *vs.* PRE with a Wilcoxon rank-sum test.

Characteristics	CTRL *n* = 260	PRE *n* = 116	DM2 *n* = 183	*p* Value
Age (years)	48.25 (41–52.5)	49.97 (43–54)	58.87 (50–64) ^a^^,b^	<0.001
Male, *n* (%)	127 (47.6)	47 (18.3)	87 (34)	0.4360
Female, *n* (%)	138 (45.54)	69 (22.77)	96 (31.6)	-
BMI (kg·m^−2^)	27.7 (25.3–29.8)	31.37 (27.5–34.4) ^a^	29.14 (26–31.4) ^a^^,b^	<0.001
SBP (mmHg)	113.9 (110–120)	117.1 (110–120)	120.6 (110–130) ^a^	<0.001
DBP (mmHg)	74.6 (70–80)	77.9 (70–80) ^a^	77.5 (70–81) ^a^	<0.001
Smoking, *n* (%)	61 (23.6)	29 (25)	35 (19.1)	0.4050
Glucose (mg·dL^−1^)	91.1 (86–97)	106.8 (99–113) ^a^	153.8 (108-169) ^a^^,b^	<0.001
HbA1c (%)	4.6 (4.2–5.1)	5.9 (5.7–6.1) ^a^	7.4 (5.8–8.4) ^a^^,b^	<0.001
HOMA-IR	1.8 (1.3–2.3)	4.8 (3.2–5.9) ^a^	-	<0.001
TG (mg·dL^−1^)	167.6 (106–210)	202 (130–242) ^a^	214.2 (126–244) ^a^	0.0059
T-C (mg·dL^−1^)	194.1 (167–217)	195.3 (168.5–218.5)	185.3 (150–212)	0.0910
HDL-C (mg·dL^−1^)	50.9 (43–58)	46.1 (39–53) ^a^	43.7 (35–55) ^a^	<0.001
LDL-C (mg·dL^−1^)	112.7 (94.5–132.5)	110.1 (90–130)	110.3 (84–129.5)	0.8800

BMI: Body mass index, SBP: systolic blood pressure, DBP: diastolic blood pressure, HOMA-IR, homeostasis model assessment of insulin resistance, HbA1c: glycated hemoglobin, TG: triglycerides, T-C: total cholesterol, HDL-C: high density lipoprotein cholesterol, LDL-C: low density cholesterol.

### 2.2. Oxidative Markers

**Figure 1:**
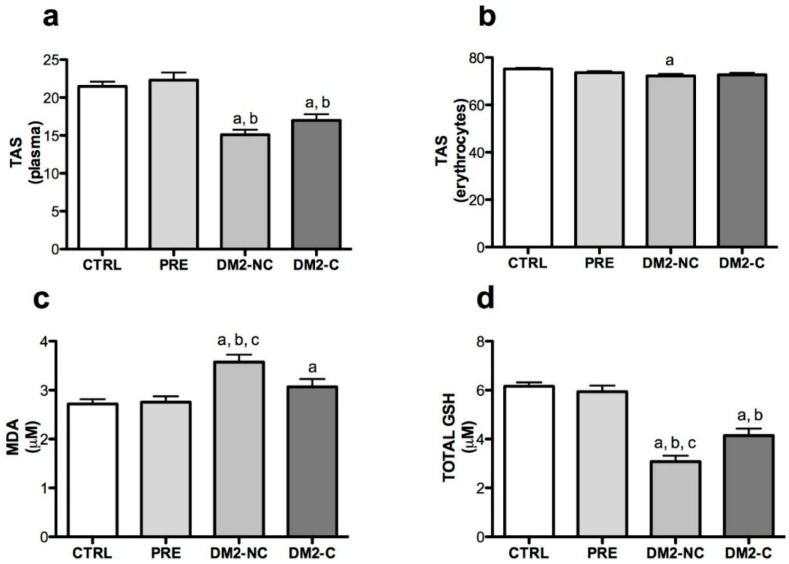

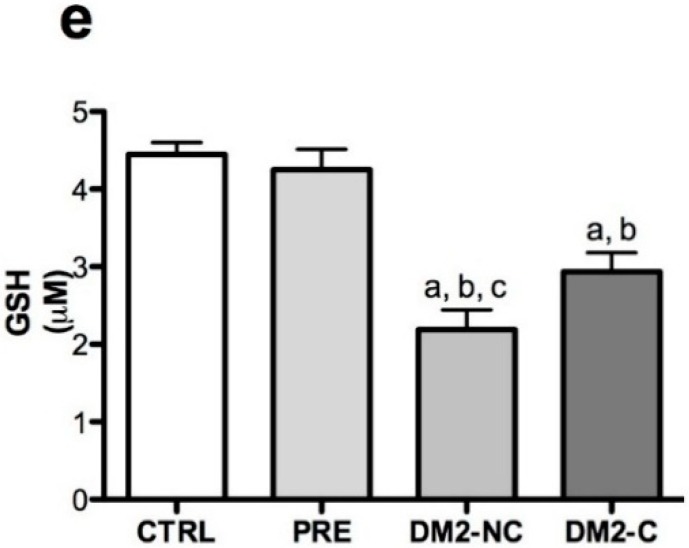
Oxidative markers in the studied groups. (**a**) Total antioxidant status (TAS) expressed as % of 2,2-diphenyl-1-picrylhydrazyl (DPPH) scavenging in plasma and (**b**) erythrocytes; (**c**) malondialdehyde (MDA) levels in plasma; (**d**) total glutathione (GSH) and (**e**) reduced form of GSH concentrations in plasma. CTRL: control, PRE: prediabetes, DM2-NC: diabetic subjects without glycemic control (glycated hemoglobin ≥ 7%), DM2-C: diabetic subjects under glycemic control (glycated hemoglobin < 7%). Each bar represents mean ± S.E. Statistical significance *p* < 0.05: ^a^ *vs.* CTRL and ^b^ *vs.* PRE ^c^ *vs.* DM2-C with a Wilcoxon rank-sum test.

To evaluate the role of glucose control in oxidative stress, the DM2 group was divided in two groups according to the HbA1c levels. DM2-NC refers to diabetic patients without glucose control (HbA1c ≥ 7%) and DM2-C which refers to diabetic patients with glucose control (HbA1c < 7%). The redox status was determined in prediabetic and diabetic individuals by measuring TAS in plasma and erythrocyte by the 2,2-diphenyl-1-picrylhydrazyl (DPPH) method (Figure 1a,b). The results showed no significant differences in oxidative markers in prediabetic individuals. TAS on plasma (DM2-NC: 15.1% ± 5.8% and DM2-C: 17.4% ± 8%, *p* < 0.001 compared *vs.* CTRL) and erythrocytes was decreased in diabetic individuals. DPPH was negatively associated with age, gender and HbA1c in plasma and just with age and HbA1c in erythrocytes (Table 2). Moreover, MDA was found higher in DM2-NC compared *vs.* CTRL (µM MDA, *p* < 0.001) but there was a lower concentration in DM2-C group compared with DM2-NC (3.07 ± 1.5 *vs.* 3.5 ± 1.5 *p*: 0.032) (Figure 1d). In fact, multiple regression showed that MDA is associated with BMI and HbA1c levels (Table 2). GSH concentrations were lower in DM2 subjects, but the DM2-C group had higher concentrations of total GSH and reduced form *vs.* CTRL and DM2-C group. GSH was associated negatively with age and HbA1c (Table 2). No significant differences were found in glutathione disulfide GSSG and GSH/GSSG ratio.

**Table 2:** Associations of significant redox status markers and antioxidant activities with age, gender, BMI, glucose and HbA1c in diabetic subjects. Multiple linear regressions in diabetic group and controls. Results are β, 95% CI, *p* of each regressor variable and *R*^2^ of each model including plasma TAS, erythrocyte DPPH, MDA, GSH and SOD activity as dependent variables.

Variables		Age	Gender	BMI	Glucose	HbA1c
Plasma TAS	β	−0.21	−2.28	0.012	−0.019	−0.98
	95% CI	−0.28 to −0.13	−3.82 to −0.19	−0.16 to 0.18	−0.037 to 0.076	−1.7 to −0.2
	*p*	<0.001	0.004	0.894	0.511	0.014
	*R*^2^	0.190	-	-	-	-
Erythocyte TAS	β	−0.26	−1.59	0.086	−0.012	−1.2
	95% CI	−0.36 to −0.17	−3.40 to 0.20	−0.11 to 0.29	−0.015 to 0.040	−1.95 to −0.44
	*p*	<0.001	0.084	0.411	0.381	0.002
	*R*^2^	0.221	-	-	-	-
MDA	β	0.006	−0.14	0.04	−0.019	0.17
	95% CI	−0.01 to 0.23	−0.43 to 0.15	0.004 to 0.074	−0.004 to 0.003	0.06 to 0.28
	*p*	0.45	0.34	0.026	0.941	0.003
	*R*^2^	0.08	-	-	-	-
GSH	β	−0.074	−0.46	0.04	0.002	−0.25
	95% CI	−0.1 to −0.04	−1.01 to 0.08	−0.02 to 0.11	−0.0007 to 0.004	−0.45 to −0.055
	*p*	<0.001	0.097	0.19	0.159	0.013
	*R*^2^	0.13	-	-	-	-
SOD-1	β	0.29	7.04	0.052	−0.005	2.99
	95% CI	0.06 to 0.51	3.1 to 10.9	−0.42 to 0.53	−0.07 to 0.06	1.14 to 4.84
	*p*	0.011	<0.001	0.831	0.89	0.002
	*R*^2^	0.124	-	-	-	-

Gender: 0 males, 1 females, BMI: body mass index, HbA1c: glycated hemoglobin, MDA: malondialdehyde, GSH: glutathione, SOD-1: Cu-Zn superoxide dismutase activity.

### 2.3. Activities of Antioxidant Enzymes

**Figure 2:**
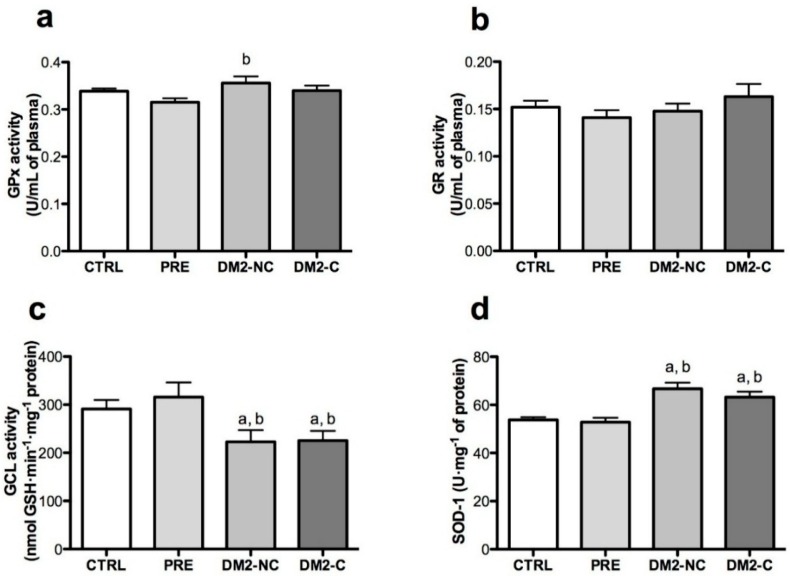
Activity of antioxidant enzymes. Plasma activity of (**a**) Glutathione peroxidase (GPx) and (**b**) Glutathione reductase (GR) and (**c**) γ-Glutamate cysteine ligase (GCL) and (**d**) Cu-Zn-superoxide dismutase (SOD) activities in erythrocytes hemolysates. CTRL: control, PRE: prediabetes, DM2-NC: diabetic subjects without glycemic control (glycated hemoglobin ≥ 7%), DM2-C: diabetic subjects under glycemic control (glycated hemoglobin < 7%). Each bar represents mean ± S.E. Statistical significance *p* < 0.05: ^a^ *vs.* CTRL and ^b^ *vs.* PRE with a Wilcoxon rank-sum test.

Activities of GPx and GR in plasma were not different in the studied groups when compared with CTRL group (Figure 2a,b). There is a trend of GPx to decrease in PRE group *vs*. DM2-NC, but not *vs.* CTRL group. There was a decrease in GCL activity in DM2-NC compared with CTRL and PRE groups (Figure 2c). The activity of GCL was adjusted by age, gender, BMI, glucose and HbA1 levels, but there was no association. SOD-1 activity was higher in DM2-NC and DM2-C when compared with CTRL group SOD-1 was associated positively with age, gender and HbA1c (Table 2).

### 2.4. Nrf2/ARE Binding Activity Assay 

**Figure 3:**
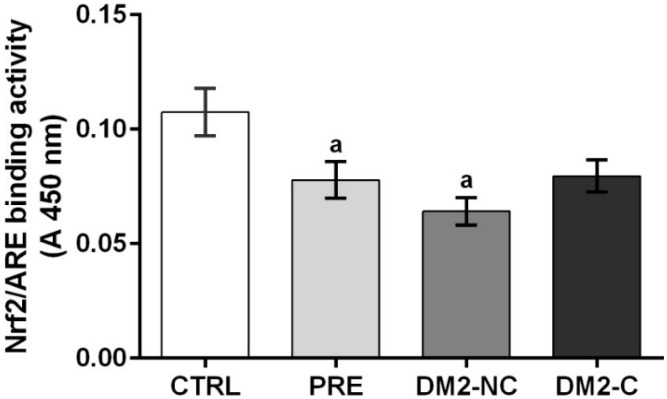
Nrf2/ARE binding activity assay: Nuclear proteins from CTRL: control subjects (*n* = 100), PRE: prediabetic subjects (*n* = 68), DM2-NC: diabetic subjects without glycemic control (Hb > 7.0%, *n* = 54), DM2-C: diabetic subjects with glycemic control (*n* = 58) were incubated with the oligonucleotide for ARE (antioxidant response element). Each bar represents mean ± S.E. ^a^ Significant difference *vs.* CTRL with *p* < 0.05.

To determine if prediabetic and diabetic status is associated with activation of Nrf2, nuclear fractions were isolated from PBMC of a representative sample of each group (100 CTRL, 68 PRE, 54 DM2-NC and 58 DM2-C). Nrf2 binding activity is decreased in PRE and DM2-NC compared with CTRL (22% and 40% of decrease *vs.* CTRL) (Figure 3). DM2-C tends to decrease (compared *vs.* CTRL and to increase compared *vs.* DM2-NC) but these differences were not significant. A regression model was applied to explain the effect of variables such as BMI, age, gender, glucose and HbA1c in prediabetic subjects and controls, however Nrf2 levels were not associated with these variables. However, there was an independent association with plasma TAS and GPx (TAS: β: −0.001, *p*: 0.047, *R*^2^: 0.024; GPx: β: 0.2, *p*: 0.008, *R*^2^: 0.05), but these associations explain less than 10% of Nrf2 variability. To explain Nrf2 variability in DM2 patients a linear regression model was made. The Nrf2 levels were transformed to logarithm scale to achieve a good fit and to avoid heteroscedasticity. The model includes glucose, HbA1c, age, gender, BMI, hospitalization by hypoglycemic events (0 = no, 1 = yes) and interaction of TAS with GSH levels. Systolic and diastolic pressure, as well as TG levels were not significantly associated in regression models and they were not included in the model. To perform this analysis, the groups of diabetics were categorized by glycemic control (DM2-C) and without control (DM2-NC) as dummy variables compared with CTRL group. HbA1c, age, hospitalization by hypoglycemic events (0 = no, 1 = yes) and interaction of TAS with GSH levels showed significant associations (Table 3).

**Table 3:** Associations of Nrf2 levels with HbA1c, age, hospitalization, diabetic groups and oxidative stress. Multiple linear regression including diabetic and control group. Results are shown as β ± S.E. of β, *p* of each regressor variable and *R*^2^ of the model including. Nrf2 data were transformed to log scale to avoid heteroscedasticity. HbA1c: glycated hemoglobin, Hospitalization: hospitalization by hypoglycemic events, 0 = no, 1 = yes, DM2-NC and DM2-C dummy variable for groups where DM2-NC include diabetic subjects without glycemic control and DM2-C include diabetic subjects with glycemic control, TAS * GSH: interaction of total antioxidant status (plasma DPPH) and reduced glutathione.

Variables	β ± S.E. of β			*p*	*R*^2^
HbA1c	−0.097	±	0.04	0.042	0.194
Age	−0.011	±	0.005	0.045	-
Hospitalization	0.48	±	0.19	0.013	-
DM2-NC	−1.06	±	0.23	<0.001	-
DM2-C	−0.48	±	0.14	0.001	-
TAS * GSH	−0.008	±	0.0004	0.044	-

## 3. Discussion

DM is an important area of investigation worldwide and represents an important disease with an increase of morbidity and mortality. Oxidative stress plays an important role in the pathogenesis of diabetic complications when glucose levels are not controlled. Since the protective role of Nrf2 in diabetes and its complications in rat models has been known, its activation has been suggested for the control of diabetes and oxidative stress induced by hyperglycemia. There are no clinical researches about the role of Nrf2 in diabetic patients.

The levels of different oxidative stress markers in plasma and erythrocytes from prediabetic and diabetic individuals were measured in the present study. Initially, our population sample was representative from Mexico in terms of BMI, because 70% of the Mexican population suffers from overweight and obesity [3]. Patients over 70 years old without diabetes are not common patients in Family Medical Clinics. Therefore, we adjusted each variable of oxidative stress with age, gender, BMI, glucose and HbA1c levels. Consistent with previous findings it was found that TAS, total GSH and reduced GSH are diminished in diabetic status, but this trend in the prediabetic group was not found. There are few clinical studies about redox status in prediabetic patients and results are controversial. Dzięgielewska-Gęsiak *et al*. [23] found diminished plasma TAS (measure by the 2,2'-azobis-3-ethylbenthiaazoline-6-sulfonic acid (ABTS) method) and an increment of MDA levels in 28 elderly prediabetic subjects. However, Al-Aubaidy and Jelinek [24] and Zengi *et al*. [25] did not find changes in GSH and lipid peroxidation in prediabetic subjects but they found an increased level of 8-hydroxy-2-deoxy-guanosine (8-OHdG), suggesting that DNA damage during prediabetes may occur before lipid peroxidation or decreased antioxidant defenses, that is observed during diabetes. These differences are probably due to the sample size and genetic factors that predispose to changes in oxidative stress during prediabetes.

A decrease in GSH levels in diabetes was reported in previous studies [26,27,28]. Total GSH and GSH levels were found decreased in the diabetic population, but the GSH/GSSG ratio was not significantly affected in the studied groups. This may be due to the fact that GPx and GR activities were not modified in any group. In addition, GCL, the enzyme that catalyzes the rate-limiting step in GSH biosynthesis [29], was significantly lower in DM2-NC subjects compared with the CTRL group. These data suggest that decreased GSH levels may be secondary to GCL activity and not by its recycling. When diabetic subjects are in glycemic control, measure by HbA1c (<7%) as indicated by the American Diabetes Association (ADA) [4], GSH levels were not strongly decreased. These results agree with Kulkarni *et al*. [30] and Pavlović *et al*. [31]; they found that GSH levels increased by treatment with sulfonylureas and biguanides. In fact, new studies focus on the use of GSH as a marker of glycemic control [30].

TAS reflects both exogenous and endogenous antioxidants in plasma. Due to the major antioxidant, GSH decreases and lipid peroxidation products measured as MDA increases, lead to diminished TAS in DM2 in plasma and erythrocytes. Oxidative stress is more evident in diabetes status than prediabetes. Recent reports suggest that oxidative damage, despite the increase in the activity of SOD [32,33] by a possible adaptive response due to ROS, is generated during diabetes. Our results agree with this hypothesis, but this point is very controversial. Other reports indicate that SOD is diminished in diabetic patients [34]. It has been suggested that the mechanism by which this occurs requires higher activity of catalase and GPx. Plasma GPx tends to be higher in the DM2-NC group, but differences were not significant and this investigation did not include catalase activity; thus further analysis is needed to elucidate this point. Another explanation may involve genetic factors that could alter the functionality and activity of these enzymes according to populations.

Our results are consistent with previous reports showing oxidative stress in diabetic patients, a novel role of Nrf2 from PBMC associated with HbA1c levels was found in this study. It is recognized that oxidative stress, inflammation and insulin resistance have a close relationship with Nrf2 in animal models. This factor up regulates the expression of enzymes that maintain cellular redox homeostasis including heme oxygenase 1 (HO-1), GPx, glutathione *S*-transferase A1, NAD(P)H quinone oxidoreductase 1 and GCL. In animal models, Nrf2 agonists improve insulin resistance and obesity in adipose tissue and prevent apoptosis in β-cells [16,17,21,35]. Nevertheless, it is not clear which tissue exerts the principal action against insulin resistance and the development of diabetes. In this study, we found lower Nrf2 activity since prediabetes, but the variables included in this study were not enough to explain Nrf2 variability during prediabetes. We just find a weak association of TAS and GPx with Nrf2, but these associations do not explain more than 5% of the Nrf2 variability during prediabetes. We speculate that the ranks of HbA1c levels need to be much closer to find a strong association with Nrf2 between PRE and CTRL groups. Also, it is known that Nrf2 is regulated by dietary compounds [36,37], but exogenous antioxidant consumption was not controlled in this study. A trial has been published on prediabetic subjects, who were randomly assigned to receive either curcumin, an activator of Nrf2, or a placebo for nine months to prevent diabetes development [38]. They found an improvement of overall function of β-cells and a diminution in the number of prediabetic individuals who eventually developed T2DM was lower. We propose that the effect of dietary compounds in Nrf2 and prediabetes maybe be a topic for future research.

A significant decrease was found of Nrf2 in DM2-NC patients. It would be expected that oxidative stress in these diabetic patients would enhance Nrf2 levels. Therefore, these findings suggest that the Nrf2 response in these patents is impaired. The regression analysis suggest that the lack of Nrf2 induction may be explained, at least in part, by the increase of HbA1c levels, by the age and by the diminution in TAS and GSH. However, these variables just explain 19% of the variability of the levels in our population sample. Lower levels of Nrf2 in individuals with poor glucose control were found. So far a mechanism by which hyperglycemia causes a decrease in the activation of Nrf2 is unknown in peripheral blood mononuclear cells. Some studies in podocytes and cardiomyocytes incubated with high glucose concentrations show that Nrf2 transcript is increased [18,39]. In contrast, in animals with chronic renal failure it was found that oxidative stress and inflammation in the kidney are compounded by conspicuous impairment of Nrf2 activation and consequent downregulation of the antioxidant enzymes [40]. However, we assume that most severe hyperglycemic states have a longer negative effect on the induction of Nrf2 by yet unknown mechanisms, coupled with an inflammatory condition that may influence the activation of Nrf2. On the other hand, single nucleotide polymorphism (SNP) of the promoter region of Nrf2 is associated with the development of different diseases that have in common the presence of oxidative stress [41,42,43,44], but so far there are no records of its association with diabetes. We suggest that SNP may be a factor that modulates the response of Nrf2 in our populations. Finally, our study has some limitations. First, mRNA levels of Nrf2-dependent enzymes and other parameters such as inflammation markers, known to be involved in Nrf2 regulation, were not measured in these patients. However, our results suggest that Nrf2 can be a target to attenuate diabetic complications induced by oxidative stress starting after the prediabetes state.

## 4. Experimental Section

### 4.1. Study Design and Subjects

The present study was conducted according to the Declaration of Helsinki. The project was approved by the Research, Ethics and Biosafety Committee of the Hospital Regional Lic. Adolfo López Mateos (registration number 253.2013). Subjects were recruited from Family Medical Clinics of the Instituto de Seguridad y Servicios Sociales de los Trabajadores del Estado (ISSSTE) in the south of Mexico city. A total of 559 Mexican individuals older than 35 years old were included. According to their FPG and HbA1c levels, individuals were classified into three groups. Normoglycemic subjects (FPG < 100 mg/dL and HbA1c < 5.7%) were referred to as Control (CTRL) group (*n* = 259). PRE group included subjects whose FPG values were of 100–125 mg/dL and HbA1c 5.7%–6.4%, known as pre-diabetic subjects. The third group consisted of previously diagnosed diabetic subjects with or without medical supervision, with over five years of disease evolution (DM2). Patients with other types of DM, alcoholic subjects, pregnant women, persons with clotting disorder and erythrocythemia were excluded. All participants gave informed consent and completed a questionnaire comprising heredofamilial diseases and medical history.

### 4.2. Sample Collection and Biochemical Analysis

Three samples (each of 6 mL) of venous peripheral blood were obtained from the antecubital vein after 8–10 h of night time fasting. One sample was collected into a serum separator tube and was centrifuged at 1500× *g* for 10 min at 4 °C. Fasting glucose levels and lipid status parameters such as T-C, LDL-C, HDL-C and TG concentrations were measured in serum using a Miura 200 autoanalyzer employing commercial kits (I.S.E., Rome, Italy). One ethylenediaminetetraacetic acid (EDTA) sample tube was used for blood collection. Ten µL of EDTA-blood were used for HbA1c quantification in a Miura 200 autoanalyzer and the remnant was used for plasma and erythrocytes isolation by centrifugation (1500× *g* for 10 min at 4 °C). The second blood EDTA tube was used for the PBMC isolation. Plasma was used to measure TAS, MDA and GSH and activity of GPx and GR. Erythrocytes were washed with cold 0.9% saline solution twice and hemolysates with double distilled (dd) H_2_O (1:5) and centrifuged at 1500× *g* for 10 min at 4 °C. Lysates from erythrocytes were used for GCL and SOD-1 activity assays.

### 4.3. PBMC and Nuclear Extract Isolations

PBMC isolation was performed on a representative sample of each group (100 CTRL, 68 PRE, 112 DM2), using a commercial kit (Lymphoprep™) from Axis-Shield (Oslo, Norway) and the procedure was conducted according to the manufacturer’s instructions. Immediately, the nuclear fraction was isolated using Nuclear Extract commercial Kit (Active Motif, Carlsbad, CA, USA). The nuclear fractions were stored at −80 °C until the Nrf2 determinations were performed.

### 4.4. Redox Status Markers

DPPH, MDA, GSH, GPx, GR and SOD-1 were measured spectrophotometrically by colorimetric assays. GCL was measured fluorophotometrically using the corresponding fluorophore. Each assay was carried out in a Multi-Mode Reader Synergy HT (Biotek, Winooski, VA, USA).

#### 4.4.1. Total Antioxidant Status (TAS)

This was measured using DPPH as previously described by Koren *et al*. [45]. The antioxidant potencies of plasma and washed erythrocytes reduce the DPPH to the yellow-colored dyphenylpricryl-hydrazine. The adduct absorbance was measured at 518 nm and the results were expressed as percentage of DPPH inhibition.

#### 4.4.2. MDA and GSH Content in Plasma

MDA concentration was measured in plasma using a standard curve of 1,1,3,3-tetramethoxypropane [46]. Total glutathione (GSH + GSSG) levels were measured in plasma using the GR enzyme method. This assay is based on the reaction of GSH which produces 5,5-dithio-bis (2-nitrobenzoic acid) (DTNB) from 5-thio-2-nitrobenzoic acid (TNB), detectable at 412 nm. The procedure was carried out as previously reported [47].

#### 4.4.3. Activity of Antioxidant Enzymes in Plasma

The activity of GPx was measured using H_2_O_2_ as substrate and GR activity was determined using GSSG as substrate and both by measuring the disappearance of NADPH at 340 nm each minute for 3 min. One unit of GPx or GR is defined as the amount of enzyme that oxidizes 1 µmol of NADPH/min. The activities of both enzymes were expressed as U/mL of plasma.

#### 4.4.4. Activity of Antioxidant Enzymes in Erythrocyte

SOD-1 activity was measured using xanthine-xanthine oxidase for generation of superoxide anion and nitroblue-tetrazolium (NBT) as indicator reagent that is reduced to formazan detectable at 586 nm. One unit of SOD-1 is defined as the amount of protein that inhibits NBT reduction by 50%. γ-GCL activity was determined according to White *et al.* [48]. When glutamate and cysteine were added to the reaction mixture, the formation of fluorescent adducts of the compound 2,3-naphthalene dicarboxaldehyde (NDA), was measured. The fluorescence was determined using an excitation and emission wavelengths of 485 and 535 nm, respectively. Results were expressed as nmol GSH synthesized above basal/min/mg erythrocyte protein. 

### 4.5. Nrf2 Binding Activity Assay

Protein concentration was determined using the Bio-Rad Protein Assay Dye from Bio Rad Laboratories (Hercules, CA, USA). A commercial kit (TransAm^®^ Nrf2, Active-Motif., Carlsbad, CA, USA) was used for the binding activity assay of Nrf2. This kit contains an immobilized oligonucleotide containing the ARE consensus-binding site (5'-GTCACAGTGACTCAGCAGAATCTG-3'). The active form of Nrf2 contained in the nuclear extract specifically binds to this oligonucleotide. Twenty µg of nuclear extract was allowed to bind to the ARE on 96-well plates. A primary antibody was used to detect Nrf2, which recognizes an epitope on Nrf2 protein upon DNA binding. A horseradish peroxidase (HRP)-conjugated secondary antibody was added to provide a sensitive colorimetric readout at 450 nm. Nuclear extracts from COS-7 cells transfected with Nrf2 were used as positive control.

### 4.6. Statistical Analyses

Statistical analyses were performed using STATA 12 (StataCorp, College Station, TX, USA). The Gaussian distribution was tested by the Shapiro-Wilk test. Analysis of variance was used to compare CTRL, PRE and DM2 subjects followed by a *post hoc* analysis (Bonferroni). A nonparametric Kruskal-Wallis test was used to evaluate variables with a non-Gaussian distribution. For evenly distributed outcomes, values were described as mean and with standard error, otherwise outcomes, were expressed as median and interquartile ranges (with presentation of 1st and 3rd quartile). Linear multiple regressions were used to estimate the relationship between redox markers or Nrf2 activity and clinical or anthropometric parameters. Graphics were made using GraphPad Prism software v. 6.0 (GraphPad Prism Inc., San Diego, CA, USA). A *p* < 0.05 was considered significant.

## 5. Conclusions

Nrf2 was diminished during prediabetic and diabetic states and oxidative stress was elevated in diabetes. Thus, these data suggest that Nrf2 decrease could precede the onset of oxidative stress during diabetes and could be a target for treatment of diabetes and its complications by improving redox status.
